# Editorial: Stress in young people: sports and relaxation techniques for self-management of stress

**DOI:** 10.3389/fspor.2025.1599062

**Published:** 2025-04-24

**Authors:** Bernardeau-Moreau Denis, Oboeuf Alexandre, Nassif Nadim

**Affiliations:** ^1^ ULR 7369 - URePSSS - Unité de Recherche Pluridisciplinaire Sport Santé Société, University of Lille, Lille, France; ^2^ UFR STAPS, I3SP, URP 3625, University of Paris Cité, Paris, France; ^3^ Department of Psychology, Education & Physical Education, Faculty of Humanities, Notre Dame University - Louaize (NDU), Zouk Mosbeh, Lebanon

**Keywords:** stress, self-management, self-esteem, sport, relaxation

**Editorial on the Research Topic**
Stress in young people: sports and relaxation techniques for self-management of stress

## Introduction

Recent surveys (1, 2) show that the unprecedented situation caused by the pandemic and the health crisis has brought about a dramatic and lasting increase in the stress experienced by young people, exacerbating mental health issues and poor eating habits (weight gain, anxiety and fears about the future). Scientific studies show that stress levels are high in the younger generations. While they highlight the negative effects of stress over time, they also look at ways to reduce it. Sports is one means that is often mentioned, as its beneficial effects help to reduce stress (3–5). Studies show that young people who regularly practice sports have lower stress levels. They are better able to control their emotions and have a higher degree of self-efficacy than more sedentary young people. Other studies also highlight how practicing relaxation can help lower perceived stress levels (6–9). The results of a study (10) show a significant reduction in psychological distress, anxiety and perceived stress in students who have taken part in a relaxation program.

### Theoretical context and questions

Certainly, a great amount of work has been done in this field, but a number of questions remain unanswered. What are the long-term effects of sports and relaxation? Can young people manage their own stress? The important question is whether there is an effect on stress management. For sports and relaxation techniques to have a long-term impact on daily life, young individuals need to appropriate how they are practiced. A process of internalization and incorporation is required to create the conditions for young people's self-empowerment. How can we help young people manage their own stress? The aim of this special issue is to explore these questions.

### Impact of sports and physical activity on health and stress

The work proposed by Aslam and Yong Bin evaluated the impact of a structured agility training study on sedentary behavior, mood and stress in adolescents aged 12–18 years in Pakistan (*N* = 100). Sedentary behavior, mood and stress levels were assessed at the beginning and after the intervention. Results show that mood improved with a reduction in tension and fatigue and an increase in vigor. Stress levels in the intervention group decreased significantly. Systematically organized and agile training was found to significantly reduce sedentary behavior while simultaneously improving the psychological well-being of adolescent populations.

The study by Majauskiene et al. aimed to determine whether participation in professional sports activities or exercise in a health or sports center was associated with indicators related to health behavior compared with no exercise. The survey included Lithuanian professional athletes (*N* = 293) who practice independently or in a sports or health center. The study examined health-related indicators: body mass index, subjective health, depressive mood, stress, sedentary behavior, sleep, alcohol consumption, smoking, and overeating. This study reveals that participants who engage in physical activity generally score higher on various health-related scales than those who are inactive.

This study by Hajj et al. aimed to examine the impact of mindfulness practices on stress levels and their effect on engagement in physical activity among young people. Quantitative data were collected electronically from students at the University of Rouen and the Université de Paris-Cité (*N* = 218). The questionnaires included personalized tools on sports practices and mindfulness and the Perceived Stress Scale (PSS). The results confirm the idea that integrating wellness practices into educational settings can improve the mental resilience and overall well-being of students, providing them with the essential tools to effectively manage future stressors.

### Toward empowerment and self-control through sports and physical activity

In the article by Coquinos et al., the objective was to evaluate the relationship between physical activity and the concept of the physical self in women with endometriosis aged less than 30 years (*N* = 198). The authors observed an amelioration in mental well-being and a reduction in the risk of anxiety and depression. Their results show that women who practice regular physical activity have a significantly higher self-concept than those who do not. Physical activity was found to contribute to the development of the concept of the physical self and increases the ability to overcome life's obstacles.

The objective of the study conducted by Dugué et al. was to test the effectiveness of an emotional intelligence program with health education students (*N* = 68). The authors showed that the early implementation of this program prepares students to face difficult situations through the integration of emotionally intelligent behaviors, such as reflection, Control of discomfort and appropriate expression of emotions. Their results confirm that a cooperative dynamic increases motivation and improves self-efficacy and social behaviors. According to the authors, emotional intelligence can be improved with physical activities and opens possibilities to include an emotional intelligence program in a health training program.

Trpkovici et al. aimed to show through their research how stress conditions created in virtual reality generate psychological responses in athletes compared to the responses they experience during a competitive match. All participants (*N* = 24 female athletes) completed the Athlete Anxiety Questionnaire in order to measure anxiety in high-stakes situations and assess levels of concentration and self-confidence during games. The results of this research indicate that the virtual reality simulated sports stress scenario triggers athletes' stress responses that are comparable to those experienced in real competitive games. Based on these results, the authors conclude that virtual reality technology shows promise as a tool for improving athletes' stress management skills and could be an important asset in sports psychology preparation processes.

## Conclusion

The articles in this special issue examined the impact of sports, physical activity and relaxation on stress in the population (women, adolescents, students, and young people). Among the concepts discussed are self-concept and mindfulness practices, which were used to measure the effects of physical exercise on women's stress and anxiety. Several experimental contexts contributed to improving well-being and awareness, such as the emotional intelligence program to develop self-efficacy or the stress situation created in virtual reality. Another example is the structural agility training protocol, which profoundly changed the mood and stress of adolescents. All these practices and experiments underlined the importance and relevance of incorporating wellness practices to combat stress and anxiety.
